# Association of Tumor Necrosis Factor-α(*TNF-α*) *-308G>A* and *-238G>A* Polymorphisms with Recurrent Pregnancy Loss Risk: A Meta-Analysis

**DOI:** 10.22074/ijfs.2019.5454

**Published:** 2018-10-02

**Authors:** Fereshteh Aslebahar, Hossein Neamatzadeh, Bahare Meibodi, Mojgan Karimi-Zarchi, Razieh Sadat Tabatabaei, Mahmood Noori-Shadkam, Mahta Mazaheri, Reihaneh Dehghani-Mohammadabadi

**Affiliations:** 1 Department of Obstetrics and Gynecology, Semnan University of Medical Sciences, Semnan, Iran; 2 Mother and Newborn Health Research Center, Shahid Sadoughi University of Medical Sciences, Yazd, Iran; 3 Department of Medical Genetics, Shahid Sadoughi University of Medical Sciences, Yazd, Iran; 4 Department of Obstetrics and Gynecology, Shahid Sadoughi University of Medical Sciences, Yazd, Iran

**Keywords:** Meta-Analysis, Miscarriage, Pregnancy Loss, Polymorphism, Tumor Necrosis Factor-α

## Abstract

**Background:**

Multiple studies have been carried out examining the association of tumor necrosis factor-α gene (*TNF-α*) promoter region polymorphisms with recurrent pregnancy loss (RPL) risk. However, the results remain con- troversial and incomplete. Hence, we performed a meta-analysis to evaluate the association of the *TNF-α -308G>A* and *-238G>A* polymorphisms with RPL risk.

**Materials and Methods:**

In this meta-analysis, a comprehensive search of PubMed, Web of Knowledge and EM- BASE was performed to identify relevant studies published until December 1, 2017. The associations were assessed by odds ratio (OR) and its corresponding 95% confidence interval (CI).

**Results:**

A total of 29 case-control studies, comprising 20 studies on *TNF-α -308G>A* (3,461 cases and 3,895 con- trols) and nine studies on *TNF-α -238G>A* (2,589 cases and 2,664 controls), were included in the meta-analysis. Over- all, we found *TNF-α -308G>A* to be associated with an increase in RPL risk under the homozygote (OR=1.716, 95% CI: 1.210-2.433, P=0.002) and the recessive (OR=1.554, 95% CI: 1.100-2.196, P=0.012) models. *TNF-α -238G>A* was also significantly associated with increased risk of RPL under the allele model (OR=1.554, 95% CI: 1.100-2.196, P=0.012). Stratified analysis revealed a more significant association between the *TNF-α -308G>A* polymorphism and increased RPL risk in Asians under the homozygote (OR=2.190, 95% CI: 1.465-3.274, P≤0.001), the dominant (OR=1.642, 95% CI: 1.269-2.125, P≤0.001) and the recessive (OR=1.456, 95% CI: 1.039-2.040, P=0.029) models, but not in Caucasians. A non-significant association was, however, identified between *TNF-α -238G>A* and RPL risk based on ethnicity. Moreover, *TNF-α -308G>A* and *-238G>A* polymorphisms were significantly associated with in- creased risk of RPL in high quality studies and polymerase chain reaction-restriction fragment length polymorphism (PCR-RFLP) subgroups.

**Conclusion:**

The present meta-analysis demonstrates that *TNF-α -308G>A* and *-238G>A* polymorphisms are associ- ated with an increased risk of RPL.

## Introduction

Recurrent pregnancy loss (RPL) is traditionally defined as the occurrence of three or more (≥3) consecutive pregnancy losses; however, the American Society of Reproductive Medicine (ASRM) has recently redefined RPL as two or more pregnancy losses (1, 2). It is estimated that up to 3% of fertile couples have been diagnosed with RPL (3). Moreover, RPL is accompanied by an increased risk of other pregnancy complications such as preterm birth or small for gestational age newborns (4). RPL remains one of the most important issues in reproductive medicine and there are multiple barriers to the prevention, diagnosis and treatment of it (5).

Many studies have been undertaken to identify the underlying aetiology, however, the cause of miscarriage can be identified in only 50% of cases (1, 6). Maternal age and number of previous miscarriages are two independent risk factors for a further miscarriage (7). Moreover, the known causes of RPL include chromosomal and metabolic abnormalities, uterine anomalies and immunologic factors (8). The parental carriers of balanced structural chromosomal rearrangements including balanced reciprocal and Robertsonian translocations are responsible for 2-4% of RPL cases (5-9). There is much evidence that tumor necrosis factor-α *(TNF-α)* also plays an important role in the implantation, placentation and pregnancy outcome (10).

*TNF-α*, a key pro-inflammatory cytokine, is secreted by macrophages and plays an important role in apoptotic cell death and initiating an immune response (10, 11). *TNF-α* is located on human chromosomes 6p21.3, spanning 2,762bp, and contains 4 exons (11, 12). There are several common single nucleotide polymorphisms (SNPs) in *TNF-α* which can regulate the transcription and production of *TNF-α* (12). To date, several promoter region SNPs in *TNF-α* have been reported, among which two, namely *-308G>A* (rs1800629) and -238G>A (rs361525), are most frequently studied in RPL (11-13). These polymorphisms have been shown to contribute to the susceptibility of several autoimmune conditions (9, 12, 13). Moreover, numerous studies have examined the association between *TNF-α* polymorphisms and risk of RPL; however, results have been controversial and inconclusive. These inconsistencies may be partly due to low sample sizes, false positive findings, publication bias, ethnic and geographical heterogeneity, and different characteristics among studies such as sources of controls. Meta-analysis is an important tool to obtain an unbiased estimate of the role of genetic variation in disease susceptibility (14). Thus, we conducted a meta-analysis to comprehensively evaluate the association of *TNF-α -308G>A* and *-238G>A* polymorphisms with susceptibility to RPL.

## Materials and Methods

### Search strategy

This meta-analysis was conducted and reported in accordance with the Preferred Reporting Items for Systematic reviews and Meta-Analyses (PRISMA) guidelines. All studies published up to December 1, 2017 reporting the association of the *TNF-α -308G>A* and *-238G>A* polymorphisms with RPL were identified by searching the literature in databases including PubMed, EMBASE, ISI Web of Science, Google Scholar, China National Knowledge Infrastructure (CNKI), and Wanfang. The following combination of MeSH terms and keywords was used: (‘‘tumor necrosis factor alpha’’ OR ‘‘*TNF-α*’’ OR ‘‘cachexin’’ OR ‘‘cachectin’’) AND (‘‘*-308G>A*’’ OR ‘‘rs1800629’’ OR ‘‘*-238G>A*’’ ‘‘rs361525’’) AND (‘‘recurrent pregnancy loss’’ OR ‘‘pregnancy loss’’ OR ‘‘miscarriage’’ OR ‘‘RPL’’ OR “habitual abortion’’ OR ‘‘abortion’’ OR ‘‘unexplained recurrent spontaneous abortion’’) AND (‘‘gene’’ OR ‘‘allele’’ OR ‘‘genotype’’ OR ‘‘mutation’’ OR ‘‘variant’’ OR ‘‘variation’’ OR ‘‘polymorphism’’). In addition, the reference lists of retrieved articles, reviews and previous meta-analyses were manually screened for additional studies. In the case where more than one article was published by the same author using the same case series, the study with the largest sample size was investigated. Moreover, no restrictions were placed on language, and only published studies with full-text articles were included.

### Inclusion and exclusion criteria

Studies fulfilling the following selection criteria were included in this meta-analysis: i. Has original and published data, ii. Uses case-control or cohort design, iii. Examines the associations of *TNF-α -308G>A* and *-238G>A* polymorphisms with RPL risk, and iv. Provides sufficient data for calculation of odds ratio (OR) with 95% confidence interval (CI). In addition, the following exclusion criteria were also used: i. Not relevant to *TNF-α -308G>A* and *-238G>A* polymorphisms and RPL, ii. The design is based on family or sibling pairs, iii. No usable data reported, iv. The study only involved a case population, v. Animal studies, vi. Duplicated publications, and vii. Abstracts, case-only articles, editorials, and reviews. If studies had partly overlapped subjects, only the one with the largest sample size was included.

### Data extraction

Two investigators independently reviewed full manuscripts of eligible studies, and the relevant data were extracted into predesigned data collection forms. Any discrepancy was resolved by discussion or consensus by involving a third reviewer when required. The following data were collected from each study: first Authors' surname, year of publication, country of origin, ethnicity of the study population, genotyping method, source of control groups (population- or hospital-based controls), total number of cases and controls as well as numbers of cases and controls for each *TNF-α SNP* genotype, and deviation from Hardy-Weinberg Equilibrium (HWE) of the control group. Diverse ethnicities were categorized as Asian, African, Latinos and Caucasian.

### Statistical analysis

The association of *TNF-α -308G>A* and *-238G>A* polymorphisms with RPL was estimated by ORs and their 95% CIs. Z-test was carried out to evaluate the statistical significance of pooled ORs (P<0.05 was considered statistically significant). The pooled ORs for both polymorphisms were performed under the following five genetic models: allele model (B vs. A), homozygote model (BB vs. AA), heterozygote model (BA vs. AA), dominant model (BA+BB vs. AA) and recessive model (BB vs. BA+AA). The heterogeneity of studies was assessed by using Cochran’s Q-test and the I^2^ test. A significance level of <0.10 was used to indicate heterogeneity among studies. Moreover, a high value of I^2^ indicated a higher probability of the existence of heterogeneity (I^2^=0 to 25%, no heterogeneity; I^2^=25 to 50%, moderate heterogeneity; I^2^=50 to 75%, large heterogeneity; and I^2^=75 to 100%, extreme heterogeneity). When between-study heterogeneity was found a random-effects model was performed; otherwise, a fixed-effects model (Mantel-Haenszel method) was employed. HWE of genotype distribution in the controls of included studies was conducted using an online program (http://ihg2.helmholtz-muenchen.de/cgi-bin/hw/hwa1.pl), and P<0.05 was considered as significant deviation from HWE. Stratified analyses were performed according to ethnicity, source of controls and study quality (HWE status). To validate the reliability and stability of the results, sensitivity analysis was performed with a single study in the meta-analysis being removed each time to reflect the influence of the individual data set on the pooled OR. The funnel plot was employed to examine publication bias. Egger’s regression analysis was used for re-evaluation of publication bias. The significance of the intercept was determined by the t test suggested by Egger, with P<0.10 considered as representative of statistically significant publication bias. Funnel plots and Egger’s linear regression tests were used to provide a diagnosis of the potential publication bias. In the presence of bias, we utilized the Duval and Tweedie non-parametric ‘‘trim and fill’’ method to adjust results. The statistical analysis for this meta-analysis was performed by using the comprehensive meta-analysis (CMA) version 2.20 software (Biostat, USA) using two-sided P values.

## Results

**Fig.1:**
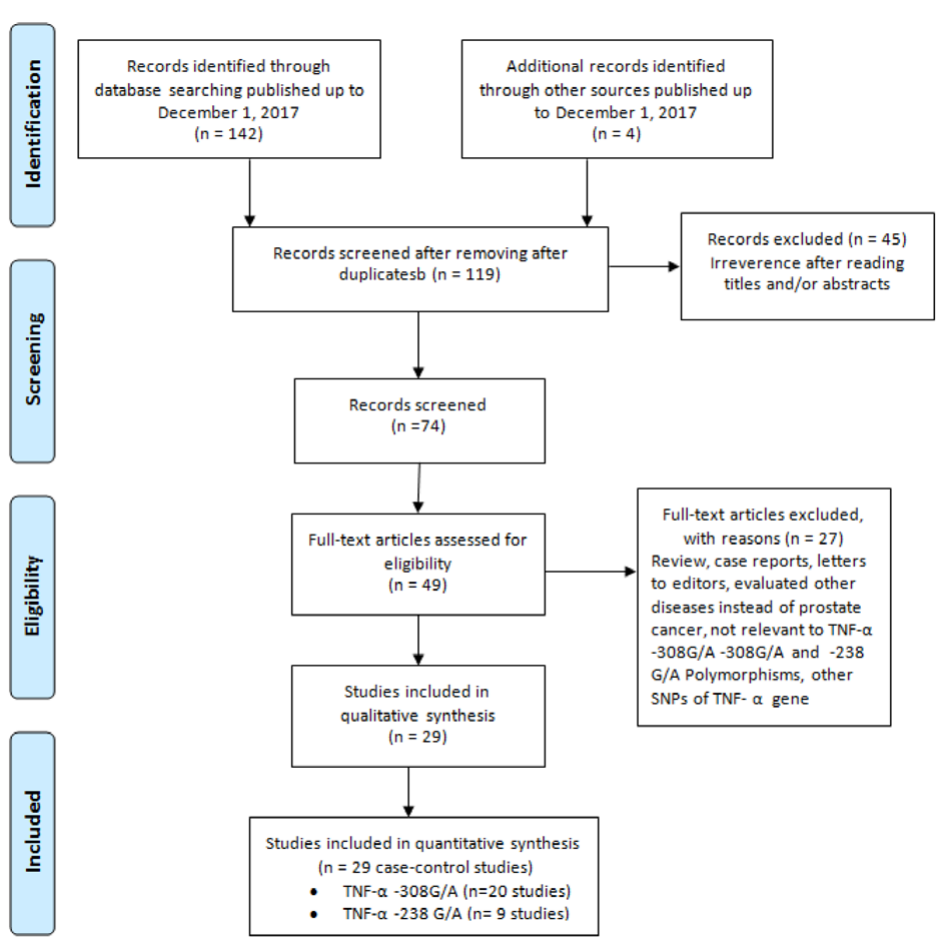
Flow chart of literature search and study selection.

The search process and search outcomes are listed in Figure 1. One hundred and forty-six potential studies were collected by database and manual search. After screening titles and abstracts, 30 studies were considered duplicates and were excluded. Following the full-text review, 45 articles were excluded as they were reviews, case reports, not case-control designed, contained no data concerning RPL and two lacked any data concerning the polymorphisms of interest. Moreover, we also examined recent reviews and meta-analyses; no additional relevant study was found. Finally, 29 case-control studies in 20 publications with 5,050 RPL cases and 6,559 controls were included in the meta-analysis. There were 20 case-control studies (15-34) for the *TNF-α -308G>A* polymorphism including 3,461 RPL cases and 3,895 controls, and nine case-control studies (25-28, 30-32, 34) for the *TNF-α -238G>A* polymorphism including 2,589 RPL cases and 2,664 controls (Table 1). Data from studies published between 2001 and 2017 were pooled to the meta-analysis. Among the 29 case-control studies, there were eight studies of Caucasians, 16 studies of Asians, two studies of Latinos and two studies of Africans. Studies had been carried out in United Kingdom, Brazil, Argentina, Germany, Iran, Mexico, Tunisia, Italy, China, Bahrain, India, Korea, and Saudi Arabia. The *TNF-α* polymorphisms were genotyped by five methods including polymerase chain reaction-amplification refractory mutation system (PCR-ARMS), PCR-restriction fragment length polymorphism (PCR-RFLP), sequence-specific oligonucleotide (SSO), PCR-sequence-specific primer (PCR-SSP) and direct sequencing. The distribution of genotypes in the controls of five studies (20, 26, 27, 32, 33) deviated from HWE expectations (P<0.05). The detailed characteristics of each study and genotype distributions included in the meta-analysis are presented in Table 1.

### Quantitative data synthesis

#### *TNF-α-308G>A* polymorphism

**Fig.2:**
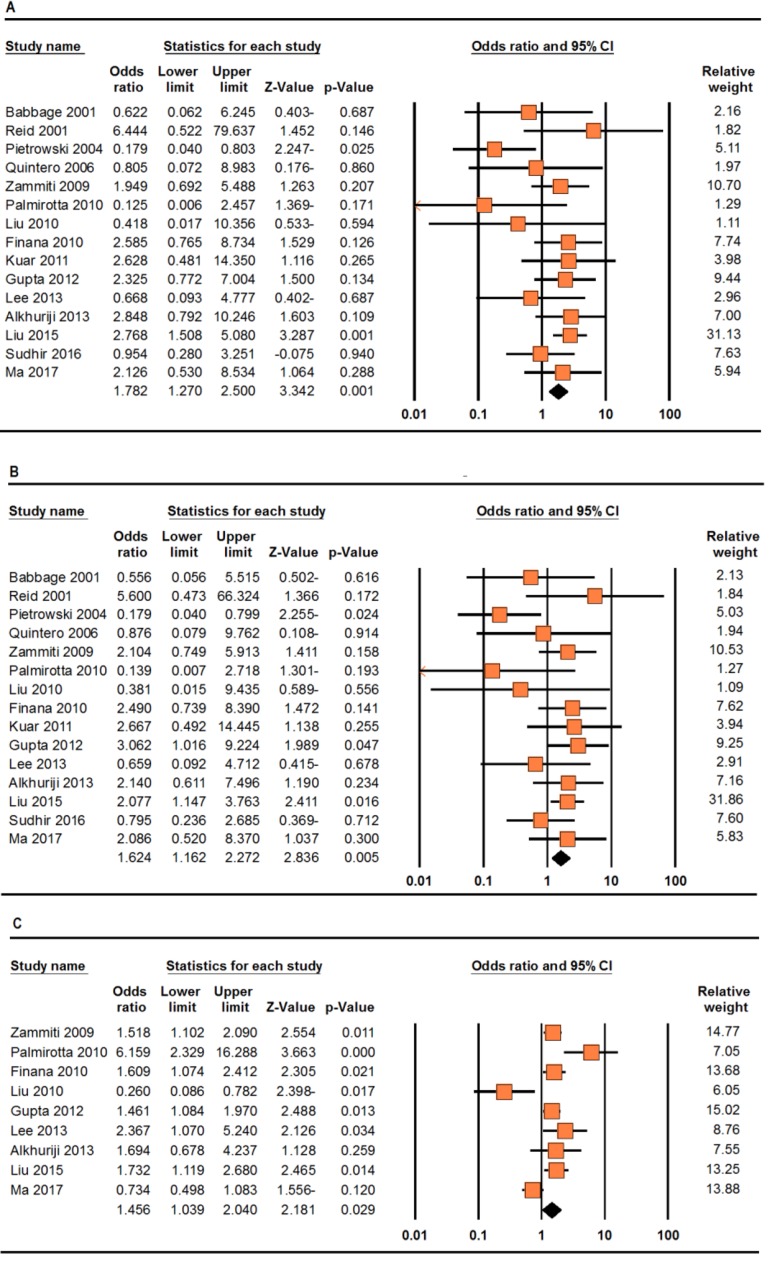
Forest plots for the association of the *TNF-α -308G>A* and *-238G>A* polymorphisms with Recurrent pregnancy loss (RPL) susceptibility. A. *TNF-α -308G>A* (homozygote model: AA vs. GG), B. *TNF-α -308G>A *(recessive model: AA vs. AG+GG), and C. *TNF-α -238G>A* (allele model: A vs. G).

Table 2 lists the main results of the meta-analysis of *TNF-α -308G>A* polymorphism and RPL risk. When all the eligible studies were pooled into the meta-analysis, a significant association was found under the homozygote model (AA vs. GG: OR=1.716, 95% CI: 1.210-2.433, P=0.002, Fig .2A) and the recessive model (AA vs. AG+GG: OR=1.554, 95% CI: 1.100-2.196, P=0.012, Fig .2B). In addition, significant between-study heterogeneity was detected for all genetic models.

When stratified by ethnicity, a significant association with increased RPL risk was observed among Asians under the homozygote genetic model (AA vs. GG: OR=2.190, 95% CI: 1.465-3.274, P≤0.001), the dominant model (AA+AG vs. GG: OR=1.642, 95% CI: 1.269-2.125, P≤0.001) and the recessive model (AA vs. AG+GG: OR=1.901, 95% CI: 1.279-2.828, P=0.002), but not among Caucasian populations. Interestingly, stratified analysis according to genotyping technique revealed a significantly increased risk of RPL in those studies using PCR-RFLP under the homozygote genetic model (AA vs. GG: OR=1.828, 95% CI: 1.253-2.667, P=0.002), the dominant model (AA+AG vs. GG: OR=1.387, 95% CI: 1.016-1.892, P=0.039) and the recessive model (AA vs. AG+GG: OR=1.666, 95% CI: 1.147-2.421, P=0.007). Subgroup analysis of studies with high quality data did not show a significant association between this polymorphism and increased risk of RPL. Summary results of different comparisons are listed in Table 2.

**Table 1:** Main characteristics of studies included in this meta-analysis


First author (Y)	Country	Genotyping	SOC	Case/ Control	Cases						Controls						MAFs	HWE
	(Ethnicity)	method			Genotype				Allele		Genotype				Allele			

**TNF-α -308G>A**					GG	AG	AA		G	A	GG	AG		AA	G	A		
Babbage et al. (15)	UK (Caucasian)	PCR-ARMS	HB	43/73	30	12	1		72	14	56	14		3	126	20	0.137	0.106
Reid et al. (16)	UK (Caucasian)	PCR–RFLP	HB	17/43	9	6	2		24	10	29	13	1		71	15	0.174	0.744
Baxter et al. (17)	UK (Caucasian)	SSO	NR	76/138	51	25(AG+AA)			-	-	94	44(AG+AA)			-	-	NA	NA
Daher et al. (18)	Brazil (Caucasian)^*^	PCR-SSP	NR	48/108	36	12(AG+AA)			-	-	89	19(AG+AA)			-	-	NA	NA
Prigoshin et al. (19)	Argentina(Caucasian)	PCR-SSP	NR	41/54	35	6(AG+AA)			-	-	4	5(AG+AA)			-	-	NA	NA
Pietrowski et al. (20)	Germany(Caucasian)	PCR–RFLP	PB	168/222	133	33	2		299	37	167	41		14	375	69	0.155	≤0.001
Kamali-Sarvestani et al. (21)	Iran (Asian)	ASO-PCR	PB	131/143	117	14	0		248	14	122	21		0	265	21	0.073	0.343
Quintero-Ramos et al. (22)	Mexico (Latinos)	PCR-SSP	PB	122/214	113	8	1		234	10	182	30		2	394	34	0.079	0.543
Zammiti et al. (23)	Tunisia (African)	PCR–RFLP	PB	372/274	319	39	14		677	67	222	47		5	491	57	0.104	0.186
Palmirotta et al. (24)	Italy (Caucasian)	Sequencing	HB	100/100	87	13	0		187	13	76	21		3	173	27	0.135	0.313
Liu et al. (25)	China (Asian)	Sequencing	HB	132/152	110	22	0		242	22	138	13		1	289	15	0.049	0.276
Finan et al. (26)	Bahrain (Asian)	PCR–RFLP	PB	204/248	164	32	8		360	48	212	32		4	447	49	0.080	0.040
Kaur et al. (27)	India (Asian)	PCR–RFLP	PB	50/50	39	6	5		84	16	41	7		2	89	11	0.110	0.043
Gupta et al. (28)	India (Asian)	PCR–RFLP	PB	300/500	229	62		9	520	80	425	70		5	920	80	0.080	0.274
Bompeixe et al. (29)	Brazil (Caucasian)^*^	PCR-SSP	NR	61/75	45	16(AG+AA)			-	-	16(AG+AA	59			-	-	NA	
Lee et al. (30)	Korea (Asian)	PCR–RFLP	PB	357/236	319	36	2		674	40	213	21		2	447	25	0.053	0.082
Alkhuriji et al. (31)	Saudi (Asian)	PCR-SSP	NR	65/65	33	24	8		108	22	47	14		4	90	40	0.169	0.059
Liu et al. (32)	China (Asian)	PCR–RFLP	NR	284/284	144	105	35		393	175	205	61		18	471	35	0.170	≤0.001
Sudhir et al. (33)	India (Asian)	PCR–RFLP	HB	115/111	76	34	5		186	44	87	18		6	192	30	0.135	0.001
Ma et al. (34)	China (Asian)	PCR–RFLP	PB	775/805	683	86	6		1452	98	726	76		3	1528	82	0.050	0.506
**TNF-α -238G>A**					GG	AG	AA		G	A	GG	AG		AA	G	A		
Zammiti et al. (23)	Tunisia (African)	PCR–RFLP	PB	372/274	267	88	20		616	128	215	52		7	482	66	0.120	0.084
Palmirotta et al. (24)	Italy (Caucasian)	Sequencing	HB	100/100	84	16	0		184	16	94	6		0	194	6	0.030	0.757
Finan et al. (26)	Bahrain(Asian)	PCR–RFLP	PB	204/248	148	52	4		348	60	200	48		0	448	48	0.096	0.091
Liu et al. (25)	China (Asian)	Sequencing	HB	132/152	128	4	0		260	4	135	17		0	287	17	0.055	0.465
Gupta et al. (27)	India (Asian)	PCR–RFLP	PB	300/500	121	63	16		509	91	154	113		31	891	109	0.293	0.138
Lee et al. (30)	Korea (Asian)	PCR–RFLP	PB	357/236	330	26	1		686	28	228	8		.0	464	8	0.016	0.791
Alkhuriji et al. (31)	Saudi (Asian)	PCR-SSP	NR	65/65	55	7	3		117	13	57	8		0	122	8	0.016	0.597
Liu et al. (32)	China (Asian)	PCR–RFLP	NR	284/284	240	30	14		510	58	249	35		0	533	35	0.016	0.268
Ma et al. (34)	China (Asian)	PCR–RFLP	PB	775/805	732	41	2		1505	45	745	57		3	1547	63	0.039	0.097


*; Authors declared that the ancestry of the participants was European (Caucasians). ARMS-PCR, Amplification refractory mutation system-polymerase chain reaction, RFLP; Restriction fragment length polymorphism, SSO; Sequence-specific oligonucleotide, SSP; Sequence-specific primer, ASO-PCR; Allele-specific polymerase chain reaction; SOC; Source of controls, HB; Hospital based, PB; Population based, NR; Not reported, MAFs; Minor allele frequencies, HWE; Hardy-weinberg equilibrium, and NA; Not applicable.

**Table 2:** Results of meta-analysis for the association of the TNF-α -308G>A polymorphism and risk of RPL


Subgroup	Genetic model	Type of model	Heterogeneity			Odds ratio (OR)			Publication bias	
			I^2^ (%)	P_H_	O_R_	95% CI	Z_test_	P_OR_	P_Beggs_	P_Eggers_

Overall	A vs. G	Random	87.66	≤0.001	1.151	0.805-1.646	0.769	0.442	0.964	0.296
	AA vs. GG	Fixed	31.51	0.117	1.782	1.270-2.500	3.342	0.001	0.198	0.038
	AG vs. GG	Random	52.75	0.009	0.699	0.411-1.190	-1.319	0.187	0.443	0.956
	AA+AG vs. GG	Random	66.08	≤0.001	1.235	0.981-1.554	1.797	0.072	1.000	0.470
	AA vs. AG+GG	Fixed	27.21	0.156	1.624	1.162-2.272	2.836	0.005	0.092	0.084
By ethnicity										
Caucasian	A vs. G	Random	63.68	0.041	0.859	0.492-1.498	-0.537	0.591	0.308	0.416
	AA vs. GG	Fixed	53.96	0.089	0.416	0.145-1.190	-1.66	0.102	0.734	0.562
	AG vs. GG	Random	85.78	≤0.001	0.540	0.042-7.007	-0.471	0.637	0.734	0.873
	AA+AG vs. GG	Fixed	26.01	0.230	0.990	0.759-1.291	-0.077	0.939	0.071	0.198
	AA vs. AG+GG	Fixed	50.44	0.109	0.407	0.143-1.158	-1.685	0.092	0.734	0.564
Asian	A vs. G	Random	90.54	≤0.001	1.543	0.880-2.706	1.514	0.130	0.536	0.335
	AA vs. GG	Fixed	0.00	0.654	2.190	1.465-3.274	3.822	≤0.001	0.265	0.071
	AG vs. GG	Fixed	0.00	0.532	0.885	0.557-1.314	-0.713	0.476	0.386	0.617
	AA+AG vs. GG	Fixed	47.93	0.052	1.642	1.269-2.125	3.771	≤0.001	0.754	0.224
	AA vs. AG+GG	Fixed	0.00	0.632	1.901	1.279-2.828	3.174	0.002	0.265	0.243
Genotyping technique										
PCR-RFLP	A vs. G	Random	90.14	≤0.001	1.516	0.917-2.507	1.622	0.105	1.000	0.708
	AA vs. GG	Fixed	46.06	0.062	1.828	1.253-2.667	3.130	0.002	0.602	0.325
	AG vs. GG	Random	55.32	0.022	0.760	0.390-1.482	-0.804	0.421	0.916	0.717
	AA+AG vs. GG	Random	67.69	0.002	1.387	1.016-1.892	2.061	0.039	0.754	0.434
	AA vs. AG+GG	Fixed	43.51	0.078	1.666	1.147-2.421	2.677	0.007	0.602	0.513
Studies quality (HWE)										
High quality studies	A vs. G	Random	77.52	≤0.001	0.974	0.620-1.528	-0.116	0.908	1.000	0.254
	AA vs. GG	Fixed	7.84	0.370	1.561	0.821-2.970	1.358	0.174	0.536	0.074
	AG vs. GG	Random	54.98	0.030	0.514	0.188-1.406	-1.296	0.195	0.265	0.418
	AA+AG vs. GG	Random	53.50	0.011	1.208	0.904-1.616	1.276	0.202	1.000	0.504
	AA vs. AG+GG	Fixed	9.35	0.358	1.584	0.836-2.999	1.411	0.158	0.265	0.046


RPL; Recurrent pregnancy loss, PCR-RFLP; Polymerase chain reaction-restriction fragment length polymorphism, CI; Confidence interval, and HWE; Hardy-weinberg equilibrium.

#### *TNF-α -238G>A* polymorphism

Table 3 lists the main results of the meta-analysis of *TNF-α -238G>A* polymorphism and RPL risk. When all the eligible studies were pooled into the meta-analysis, a significant association was found under the allele model (A vs. G: OR=1.456, 95% CI: 1.039-2.040, P=0.029, Fig .2C). Interestingly, when stratified by ethnicity, there was no significant association with an increased RPL risk in the Asian population. Stratified analysis according to genotyping technique revealed a significantly increased risk of RPL in those studies involving PCR-RFLP under the allele model (A vs. G: OR=1.418, 95% CI: 1.077-1.867, P=0.013).

### Minor allele frequencies

The minor allele frequencies (MAFs) of the *TNF-α -308G>A* and *-238G>A* polymorphisms are presented in Table 1. The MAF for *TNF-α-238G>A* polymorphism was ranged between 3.0-29.3% in overall population. However, the allele and genotype distributions of *TNF-α -308G>A* polymorphism showed ethnic variation. The MAF in the Asian and Caucasian populations were 10.95% (4.9-17.0%) and 15.45% (13.5-17.4%) respectively, showing a lower frequency in Asians (Table 1).

### Test of heterogeneity and sensitivity analyses

There was significant heterogeneity among these studies for the *TNF-α -308G>A* polymorphism under the allele model (A vs. G: P_H_≤0.001), the heterozygote model (AG vs. GG: P_H_=0.009) and the dominant model (AA+AG vs. GG: P_H_≤0.001). Then, we assessed the source of heterogeneity by meta-regression analysis. Surprisingly, ethnicity, glaucoma types, genotyping methods and study quality did not contribute to substantial heterogeneity in the meta-analysis (Table 2).

**Table 3:** Results of meta-analysis for the association of the *TNF-α -238G>A* polymorphism and risk of RPL


Subgroup	Genetic model	Type of model	Heterogeneity			Odds ratio (OR)			Publication bias	
			I^2^ (%)	P_H_	O_R_	95% CI	Z_test_	P_OR_	P_Beggs_	P_Eggers_

Overall	A vs. G	Random	74.99	≤0.001	1.456	1.039-2.040	2.181	0.029	0.348	0.801
	AA vs. GG	Random	59.35	0.022	2.134	0.792-5.751	1.498	0.134	1.000	0.088
	AG vs. GG	Random	68.43	0.001	1.051	0.746-1.482	0.284	0.776	0.754	0.820
	AA+AG vs. GG	Random	68.61	0.001	11.94	0.864-1.652	1.073	0.283	1.000	0.705
	AA vs. AG+GG	Fixed	48.85	0.068	1.374	0.865-2.184	1.345	0.179	0.763	0.084
By Ethnicity										
Asian	A vs. G	Random	73.09	0.001	1.269	0.872-1.848	1.245	0.213	1.000	0.711
	AA vs. GG	Random	58.47	0.034	2.420	0.640-9.154	1.302	0.193	0.707	0.039
	AG vs. GG	Random	64.64	0.009	0.906	0.625-1.316	-0.517	0.605	1.000	0.950
	AA+AG vs. GG	Random	67.71	0.005	1.056	0.733-1.523	0.293	0.769	0.763	0.919
	AA vs. AG+GG	Fixed	51.39	0.068	1.151	0.667-8.131	1.411	0.158	0.707	0.064
Genotyping technique										
PCR-RFLP	A vs. G	Random	64.12	0.016	1.418	1.077-1.867	2.491	0.013	0.060	0.630
	AA vs. GG	Random	62.66	0.020	1.920	0.679-5.428	1.231	0.218	0.707	0.165
	AG vs. GG	Random	65.63	0.012	1.070	0.773-1.481	0.409	0.683	0.259	0.341
	AA+AG vs. GG	Random	65.67	0.012	1.213	0.895-1.643	1.244	0.214	0.452	0.232
	AA vs. AG+GG	Fixed	52.34	0.062	1.318	0.825-2.107	1.156	0.248	0.452	0.164


RPL; Recurrent pregnancy loss, PCR-RFLP; Polymerase chain reaction-restriction fragment length polymorphism, and CI; Confidence interval.

Additionally, we performed sensitivity analysis to confirm the stability and reliability of our results by sequentially omitting individual eligible studies. When any single study was excluded, the corresponding ORs were not considerably changed, indicating the stability of the estimated OR. In addition, we excluded the studies in which genotype distribution in the controls deviated from HWE expectations, and in the homozygote and recessive models, the studies were found to affect the corresponding pooled ORs with heterogeneity removed under the recessive model (Table 2). The supplementary sensitivity analysis thus showed that the results of the present meta-analysis are reliable.

### Publication bias

**Fig.3:**
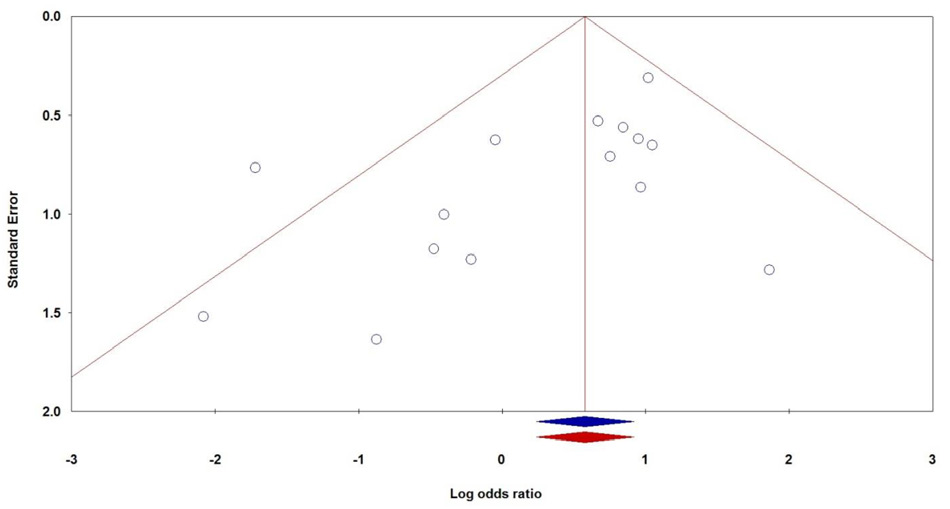
Begg’s funnel plots of the *TNF-α -308G>A* polymorphism and RPL susceptibility under the homozygote model for publication bias test (blue without and red with trim and fill test).

We used the funnel plot and Egger’s linear regression to assess publication bias of the included studies. The shapes of the funnel plots indicated no obvious asymmetry (Fig .3). The results of Egger’s test also showed no strong statistical evidence of publication bias (Table 2). However, Egger’s test found evidence for the publication bias between *TNF-α -308G>A* polymorphism and RPL risk under the homozygote model (AA vs. GG: P_Begg’s_=0.273, P_Egger’s_=0.046, Fig .3B). Therefore, we used the Duval and Tweedie non-parametric ‘‘trim and fill’’ method to adjust for publication bias. However, the results with and without ‘‘trim and fill’’ did not lead to different conclusions, indicating that our results are statistically robust.

## Discussion

Although RPL is one of the most common public health issues, little is known regarding genetic its susceptibility factors (35). Genetic variants in *TNF-α* have been associated with the etiology of RPL (9). Several important polymorphisms in the promoter region of *TNF-α* have been identified, such as the -308G>A and -238 G>A variants (27-34). Hitherto, the associations between polymorphisms in the promoter region of *TNF-α* and the risk of RPL have remained inconclusive. Thus, we performed a CMA to evaluate the association of *TNF-α -308G>A* polymorphisms with risk of RPL. The meta-analysis found a significant association between the *TNF-α -308G>A* polymorphism and RPL under the homozygote and recessive models. Moreover, to the best of our knowledge, this meta-analysis is the first to evaluate the association of *TNF-α -238G>A* polymorphism with RPL. The results of our meta-analysis revealed that the *TNF-α -238G>A* polymorphism was associated with the increased risk of RPL under the allele model. However, due to the limited number of available published studies on the *TNF-α -238G>A* polymorphism, further studies with larger sample sizes are needed to reach a more convincing conclusion.

The subgroup analyses revealed a significant association between *TNF-α -308G>A* polymorphism and RPL in Asian populations under the three genetic models. However, for Caucasians, the results indicated that this polymorphism is not associated with increased risk of RPL. The inconsistency of subgroup analysis with pooled estimates may be due to genetic diversity in different ethnicities. Furthermore, given that RPL is a multifactorial condition, beside genetic factors, endogenous and exogenous factors play a major role in RPL aetiology. Similarly, the included studies in this meta-analysis differed in their findings with regard to the association between *TNF-α -308G>A* polymorphism and risk of RPL. It is therefore possible that the difference between the studies may reflect the different ethnicities been investigated, because geographical regions may have different genetic and environmental factors that might affect the findings. Thus, this discrepancy might also be due to other factors such as maternal cigarette smoking, caffeine consumption, alcohol consumption, maternal age, number of previous miscarriages, diabetes mellitus, infective agents, endocrine factors, uterine anatomic abnormalities and antiphospholipid antibody syndrome (APS). Therefore, the relationship between this polymorphism and RPL might vary by ethnicity.

Recently, a meta-analysis by Li et al. (9) with 1,430 RPL cases and 1,727 healthy controls was performed to investigate the associations of *TNF-α* polymorphisms with RPL. Their results suggested that the *TNF-α -308G>A* polymorphism was associated with increased RPL risk. Our results are consistent with their meta-analysis. However, their meta-analysis generated contradictory results due to insufficient power because the number of studies was considerably smaller than that needed to achieve robust conclusions. In addition, due to small size, they could not rule out the possibility that publication bias was undetected. More recently, two new epidemiological studies (33, 34) have been performed to estimate the effect of the *TNF-α -308G>A* polymorphism on RPL risk in Asian populations. Moreover, we found that Li et al. had overlooked seven studies on the *TNF-α -308G>A* polymorphism and RPL risk in their meta-analysis. Hence, it may significantly affect their total results and RPL results. In the present meta-analysis, by including more nine case-control studies, we found that the *TNF-α -308G>A* polymorphism was associated with risk of RPL.

Between-study heterogeneity and publication bias are important issues that cannot be ignored in a meta-analysis (36, 37). In addition, between-study heterogeneity might distort the conclusion of a meta-analysis (38). The study designs, source of controls subjects, ethnicity, genotyping method, sample size and other variables may contribute to the heterogeneity (37-40). Here, we detected moderate between-study heterogeneity across studies under the allele, heterozygote and the dominant models. We thus selected the random-effects model to summarize the ORs. We performed meta-regression analysis to find the source of between-study heterogeneity. However, after the subgroup analysis by ethnicity, heterogeneity still remained in Caucasians. In contrast, in the Asians, there were no heterogeneity, indicating that heterogeneity could be partly accounted for by the genetic distribution of different ethnicities between continents. We observed publication bias for the association between the *TNF-α -308G>A* polymorphism and RPL risk under the homozygote model. After subgroup analysis by ethnicity, the publication bias disappeared in both Caucasians and Asians.

This meta-analysis has a number of limitations that should be noted. First, we could not perform further subgroup analysis by ethnicity among Caucasians and other ethnicities because of the limited number of published studies. Secondly, we strictly followed the inclusion and exclusion criteria to reduce possible selection bias. However, the Egger’s linear regression test showed little publication bias of overall analysis under the homozygote genetic model. Thirdly, the sources of literature searched were from a limited selection of electronic databases, and we failed to retrieve unpublished studies and articles written in other languages and also unpublished studies that might meet the inclusion criteria. Thus, potentially, publication bias might exist even though the funnel plots were found to be symmetrical. Fourthly, subgroup analyses regarding age, number of miscarriage, and other risk factors such as chronic disease were not conducted since the primary literature lacked sufficient data. Finally, the *TNF-α -308G>A* and *-238G>A* polymorphisms were not analyzed in combination with other polymorphisms of *TNF-α* and the effect of gene-gene interactions on RPL development was not undertaken. The relationship between polymorphisms of *TNF-α* gene and other genetic and environmental risk factors may be highly complicated, and extensive research is still required to ascertain how exactly the *TNF-α* polymorphisms affect the susceptibility of an individual to RPL.

## Conclusion

This meta-analysis confirmed the association of the *TNF-α -308G>A* and *-238G>A* promoter region polymorphisms with increased risk of RPL. Moreover, our meta-analysis suggests that *TNF-α -308G>A* is more likely to be associated with the risk of RPL in Asians than Caucasians. Further large, well-designed case-control studies are needed to confirm these findings.
